# A comparative performance analysis of the International Classification of Functioning, Disability and Health and the Item-Perspective Classification framework for classifying the content of patient reported outcome measures

**DOI:** 10.1186/s12955-021-01774-0

**Published:** 2021-04-23

**Authors:** Derek Rosa, Joy MacDermid, Dorota Klubowicz

**Affiliations:** 1 Physical Therapy, Western University, London, ON Canada; 2 Roth McFarlane Hand and Upper Limb Center, St. Joseph’s Hospital, London, ON Canada; 3 School of Rehabilitation Science, McMaster University, Hamilton, ON, Canada; 4 School of Physical Therapy, Western University, London, ON, Canada

**Keywords:** Classification, Questionnaires, Outcome assessment (health care), Health status indicators, Quality of life

## Abstract

**Background:**

Standardized coding of the content presented in patient reported outcome measures can be achieved using classification frameworks, and the resulting data can be used for ascertaining content validity or comparative analyses. The International Classification of Functioning (ICF) is a framework with a detailed conceptual structure that has been successfully utilized for such purposes through established coding procedures. The Item Perspective Classification (IPC) framework is a newly developed relational coding system that classifies the respondent perspective and conceptual domains addressed in items. The purpose of this study was to compare and describe the performance of these two frameworks when used alone, and in conjunction, for the generation of data pertaining to the content of patient reported outcome measures.

**Methods:**

Six health-related quality of life questionnaires with a total of 159 items were classified by two raters using the Item Perspective Classification framework in conjunction with the International Classification of Functioning. Framework performance indicators included: classification capacity (percent of items amenable to successful classification), coding efficiency (number of codes required to classify items), and content overlap detection (percent of items sharing identical classification codes with at least one other item). Inter-rater reliability of item coding was determined using Krippendorff's alpha.

**Results:**

Classification capacity of the IPC framework was 97%, coding efficiency 26, and content overlap detection was 95%; whereas respective values for the ICF were 68%, 114, and 58%. When used in conjunction values were 63%, 129, and 30%. Krippendorff's alpha exceeded 0.97 for all 3 classification indices.

**Conclusion:**

Inter-rater agreement on classification data was excellent. The IPC framework provided a unique classification of the respondent’s judgment during item response and classified more items using fewer categories, indicated greater content overlap across items and was able to describe the relationship between multiple concepts presented within the context of a single item. The ICF provided a unique classification of item content relating to aspects of disability and generated more detailed and precise descriptions. A combined approach provided a rich description (detailed codes) with each framework providing complementary information. The benefits of this approach in instrument development and content validation require further investigation.

## Background

Patient reported outcome (PRO) measures are typically collected using standardized questionnaires and are useful for evaluating health problems that are difficult or impossible to detect using biometric assessment methods [1–3]. However, identifying which questionnaire has the best content match for a specific purpose can be challenging. Multiple questionnaires that purport to examine the same construct may all possess satisfactory clinical measurement properties. This can make it difficult to identify a 'best performing' questionnaire based on published psychometric data [4–6]. Measures selected based on their reliability may not be valid for the purposes of specific interventions. Furthermore, the conceptual domain of measures is not always adequately defined, which means that the content validity is uncertain and difficult to assess. For example, multiple labels such as health status, quality of life (QoL) and health-related quality of life (HRQoL) are sometimes used interchangeably [4]_,_ although these terms may represent different theoretical constructs [7]. Based on this, it is evident that there is a clear need for classification frameworks that can be used to describe and quantify the content of questionnaire items to provide a rigorous, structured evaluation of the content validity of PROs.

Content classification can be important when evaluating existing PRO measures or creating items for new PRO measures. The process of determining content validity requires one to investigate the extent to which questionnaires contain relevant and important concepts within the context of a given measurement application [8]. Objective analysis of content contained within questionnaires is made possible through the use of standardized classification frameworks. Item codes generated from classification frameworks provide data that are appropriate for comparative analysis of questionnaires, scales, or subscales in order to determine their suitability for a specific purpose [4, 9]. The use of classification frameworks is important for the dependable measurement of health, to categorize different health constructs, and for the application in clinical settings such as in rehabilitation [10]. For example, in developing core sets of measures it is important to consider PRO measure content, perspectives, and the relationship between different constructs in creating a holistic, but feasible outcome measurement strategy. In clinical practice it is important to consider content and perspective of items because they will affect the clarity of communication between clinicians and respondents. A number of frameworks have been proposed for content classification of PRO measures [4, 9, 10], however none focus on what perspective the respondent is using when making decisions about their responses.

### ICF framework

The International Classification of Functioning, Disability and Health (ICF) is an interdisciplinary classification framework used to define, categorize and communicate the content of functioning, disability, and health [11, 12]. Concepts presented in questionnaire items can be classified using more than 1450 categories contained within the ICF framework to comparatively analyze item content [13]. The ICF was designed for many applications and has been used to define content in more than 100 scientific publications [14]. ICF classification called linking [4] involves identifying important concepts found in questionnaires, followed by linking these concepts to applicable ICF categories. The linking rules have evolved over time [4]. Further, the development of core outcome measure sets by international consensus serves as a “gold standard” for important content that has been used to assess different PRO measures [1–5]. ICF linking allows investigators to directly compare the content of questionnaires, and establish content validity within the context of their particular measurement application [5, 14]. The utility of the ICF as a concept classification tool stems from its ability to precisely describe concepts, make distinctions between similar concepts, and indicate where each concept belongs among a hierarchy of other concepts.

While ICF codes are useful for classifying a plethora of clinically-relevant concepts, the ICF was designed to describe functioning and disability, and as such has some shortcomings in the comprehensiveness of item content description. The following descriptive attributes of questionnaire items are difficult to classify using the ICF framework: (1) the respondent perspective used when responding to items, (2) the existence and nature of *relationships* that occur among two or more concepts presented by a single item, and (3) concepts that are vague, unspecific or poorly defined such as QoL, general health, and overall well-being [4, 5, 10, 13, 16–18] or life experiences/feelings like fear, pride or safety. According to Rosa [9], these limitations constitute loss of data pertaining to *item perspective*—a property of measures that can be classified using the Item Perspective Classification (IPC) framework.

### IPC framework

The IPC framework was developed from an evolutionary theory pertaining to the nature in which humans make evaluations and judgements [19], which may have direct application in PRO measurement since respondents are asked to make evaluations and judgements within the context of questionnaire administration. Classification using the IPC framework involves the consideration of item attributes pertaining to item perspective and standardized classification codes may be generated to compare item perspective of PRO measures [9]. Items are individually classified and contextual information pertaining to the purpose of the questionnaire and applicable stems/response categories are also taken into account.

Using the IPC framework, the type of *appraisal* is classified first. Items that inquire about one's present feelings (either in general, or toward someone or something) are classified as *emotional* appraisals, all other items are classified as eliciting *rational* appraisals. Items that elicit rational appraisals appeal to the logical mind and associated cognitive processes may include reasoned judgements or retrieval of memories pertaining to one's past feelings (either in general, or toward someone or something) [9]. This distinction between rational and emotional appraisals is theoretical, but we would expect some discrepancies between what a respondent thinks about their ability with respect to a given *bodily function* and how they feel about that level of ability [20–22]. This is due to the reason that expectations, goals, life demands, personality, environmental factors, and many other things might impact on how any given patient feels about a given level of ability. In fact, this is supported by the low to moderate correlations between body impairments and PRO measures. Accordingly, the distinction between rational and emotional appraisals of concepts may be important to make within the context of item content classification.

The second step in the item classification process using the IPC framework involves the identification of key concepts being appraised, and their corresponding classification using one of the following concept domains: inorganic, biological, social, and psychological. Vague, higher-order or poorly defined concepts that require simultaneous consideration of more than one domain are classified as being 'open-ended'—such as *overall well-being* or *QoL*. The concept domains included in the IPC framework classify concepts according to general attributes only, but resultant data may be useful for grouping discrepant items according to similarities in fundamental conceptual attributes.

The final step of the IPC classification process includes the identification and description of *relationships* that occur among two or more concepts presented within a single item. Symbols are used to classify the nature of relationships occurring among multiple concepts including: *interactive* relationships '*' (i.e. does your *physical health* impact your *social life*?), *forced choice* among two or more concepts '**/**' (i.e. how would you rate your *physical health* or *social life*?), or where no relationship in particular is appraised among two or more concepts '**,**' (i.e. how do you feel about your *physical health* and *social life*?). The concepts used in the preceding examples were identical, although the manner in which they are appraised differs according to the nature of relationships assessed in each item.

### IPC versus ICF

**Fig. 1:**
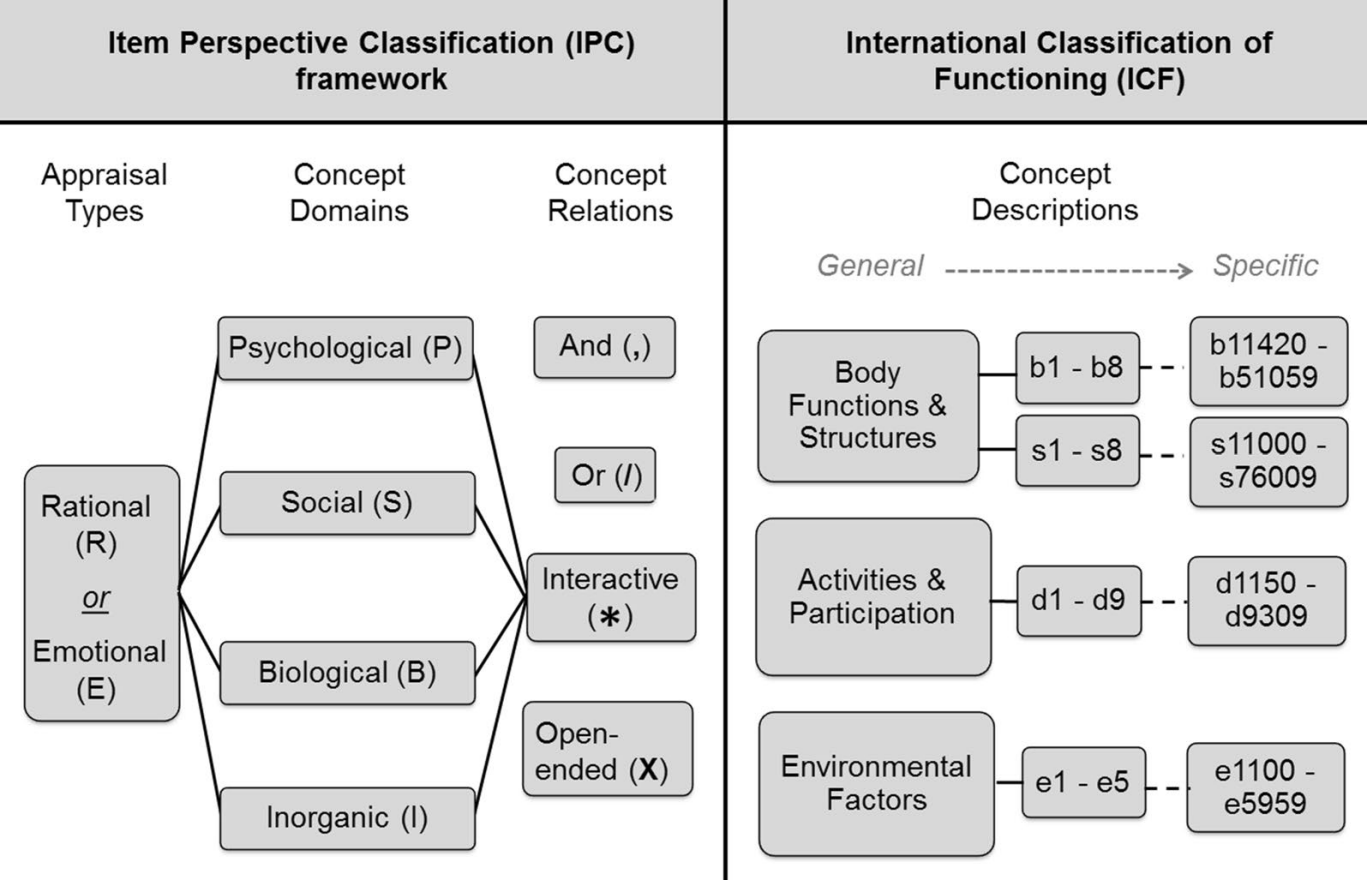
Juxtaposition of the IPC and ICF frameworks. The IPC framework classifies multiple attributes of item content including: appraisal types, concept domains and concept relations; although the description of conceptual content achieved is only at a 'general' level. The ICF framework is used solely to classify concepts contained within items, and the definition of concepts achieved may be highly specific

Differences between the ICF and IPC classification process may exist due to the inherent structural differences between these frameworks (see Fig. 1), as well as the type and amount of classification data generated from the item coding process. It is also important to consider that the level of description of *concepts* is more detailed in the ICF, since the IPC addresses content in broad terms using fewer concept categories whereas the ICF has thousands of codes arranged in a hierarchical structure that are increasingly descriptive as more detailed codes are defined. Determining the manner in which these classification frameworks perform in relation to one another has implications for the extent to which they can be considered to be suitable tools for establishing content validity of PRO measures (Fig. 1).

There are several potential benefits for using the IPC framework. For example, if the user is interested in the area of mental health, the IPC framework would be useful for identifying an applicable questionnaire that is predominantly associated within a psychological context. Although the ICF framework is useful for identifying what specific aspects of psychological functioning are mentioned in questionnaire items, there are aspects important to patients that are not fully addressed. For example, the ICF framework classifies emotional or cognitive functioning (i.e., the ability to control emotions or thought processes), but not the experience of emotions or the impact of negative thinking. Therefore, the IPC framework would better describe the nature of the issues that respondents were communicating about their life experiences. To further exemplify this, a PRO measure may have an abundance of physical/biological functioning content as classified by the ICF framework, but these codes would not reflect either the perspective taken by respondents nor the interaction between different aspects of functioning. Specifically, the IPC framework classifies whether a questionnaire asks about the impact of physiological conditions on mental health or visa versa. Furthermore, we know that ability and satisfaction with functioning can be quite different. Based on this, it is important to distinguish whether a person is rationally rating their ability, communicating a life experience, or their emotional response to their state of health. Therefore, the advantages of the IPC framework are that it is simple in terms of the number of decisions to be made, but provides a more complex description of item content. The IPC framework also provides complementary information to the ICF framework by describing the perspective taken by respondents and the relationship between different components of an item, and by classifying constructs that are not classifiable within the ICF framework. As such, the IPC framework provides a more comprehensive and nuanced assessment of content that is not possible using the ICF framework alone.

The choice of using the ICF or IPC framework alone or in combination may depend on classification purposes as well as the characteristics of the PRO measure being evaluated. Thus, it is important to determine differences in type and amount of information generated from both frameworks. Since the IPC framework may be used *in conjunction* with the ICF [9], it is also important to investigate the performance of these frameworks when used together for producing *composite item codes* that incorporate data from both frameworks. Accordingly, the purpose of this study was to compare and describe the performance of the IPC and ICF frameworks when used alone, and in conjunction, for the purpose of classifying the content of HRQoL PRO measures.

## Methods

### Sample instruments

Six HRQoL questionnaires were selected for ICF and IPC classification analysis: Medical Outcomes Study 36-Item Short Form v1 (SF-36) [23], Nottingham Health Profile (NHP) [24], the World Health Organization Disability Assessment Schedule (WHODASII) [25], European Quality of Life Instrument (EQ-5D) [26], Spitzer's Quality of Life Index (QLI) [27], and the World Health Organization Quality of Life Assessment (WHOQoL-BREF) [28]. HRQoL questionnaires were used in this investigation because they measure a construct that is inherently multidimensional, and a diversity of item content would be represented among the sample instruments. Additionally, these questionnaires vary in the type and amount of dimensions covered, mode of administration, number of items, and time required to complete the questionnaire [5]. Furthermore, these questionnaires are frequently used in literature, and have previously been used in ICF classification research [5, 17].

### Classification process

Two raters (DR, JMD) employed ICF and IPC content classification rules proposed by Rosa [9] to classify the content of N = 159 pooled items derived from the six HRQoL questionnaires mentioned above. Both raters were involved in adapting the IPC framework to conduct content analysis, and each rater had substantial experience using the ICF framework for content classification of PRO measures. To avoid poor inter-rater reliability and to achieve maximum coding agreement, raters discussed the interpretation of classification rules prior to independent coding. Collaborative item coding was subsequently conducted, which involved comparison of independent results, standardization of codes for any “rater-generated” categories assigned to items, and discussion of disagreements. Raters were not obligated to agree on any code solely for the purpose of reaching consensus. A third party was not used to settle disagreements as opposing rater opinions can be useful for the identification of poorly focused, problematic items and deficiencies within frameworks and/or classification rules.

**Fig. 2:**
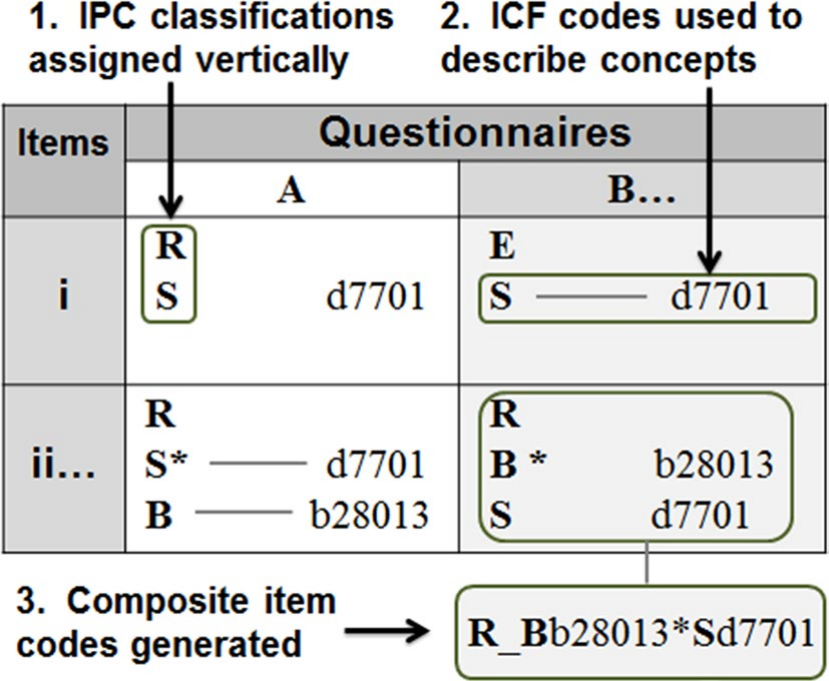
Item classification process. IPC code classification, ICF specification, and composite item code generation for the item: "Does back pain impact your family relationships?" where R = rational, S = social, B = biological, * = interaction, b28013 = back pain, d7701 = family relationships

First, all items were coded using the IPC framework and classified according to (1) the type of *appraisal* presented by an item (rational 'R' vs. emotional 'E'), (2) fundamental *domains* of concepts (inorganic 'I', biological 'B', social 'S', psychological 'P'), and (3) the existence and nature of *relationships* that occur among two or more concepts presented by a single item including interactive (*), forced choice (/), or where no declared relationship is presented (**,**) [9]. Vague, unclear, or higher-order concepts that required consideration of two or more concept domains were classified as being 'open-ended' ('X') [9]. Following IPC classification, and using established linking rules [9], ICF codes were assigned to each IPC classification to generate combined IPC + ICF codes, or *composite item codes.* These composite item codes convey information about specific concepts, the nature of the relationships between concepts, and form of appraisal. For example, using the IPC framework, the HRQoL item *"Does back pain impact your family relationships?"* would be classified as a rational appraisal ('R') of an interaction (*) between a biological concept 'B' (back pain) and a social concept 'S' (family relationships), resulting in the item code: R_B*S. These biological and social concepts can be further specified using ICF codes: *b28013* (back pain) and *d760* (family relationships). Thus, the resulting composite item code would be: R_Bb28013*S*d760* (Fig. 2).

Item concepts not amenable to classification can impact the capacity of a framework for item code generation. Therefore, similar to Cieza et al. [4, 5], rater-generated codes were used to classify concepts that were not applicable for classification using existing ICF categories. This included: (1) vague concepts that were applicable to numerous categories but did not ideally fit under one category, (2) higher-order concepts that are not clearly defined within the ICF framework such as QoL, general health, and overall well-being, and (3) difficult to classify concepts that might belong to a *personal factors* category, a component of the ICF that has yet to be coded with hierarchical alphanumeric codes and concept descriptions [15]. These unclassifiable concepts were given the prefix “**nd**” to indicate concepts “not defined” by ICF codes. For example, '**nd-gh**' was used to denote 'not defined, general health' where *general health* is a concept neither defined nor classified using existing ICF categories. Additionally, any item not applicable for classification using the IPC framework was assigned the code '**nc**' indicating that item content was 'not classified'.

### Data analysis

Differences in relative classification performance of the IPC and ICF frameworks when used alone and in conjunction were determined using descriptive statistics. *Classification capacity* (%) was calculated as the percent of sample items that: (1) contained content amenable to classification using each framework, and (2) were assigned classification codes that were agreed upon by both raters. Therefore, only items that were free from rater-generated codes and inter-rater coding disagreement were considered to be 'successfully' classified and used for the final determination of classification capacity.

In addition to percent agreement, Krippendorff's alpha (α) was used to provide an estimate of inter-rater reliability in the assignment of framework codes to sample items [29, 30] varying from < 0 (when disagreements are systematic) to 0.00 (where reliability is absent) to 1.00 (perfect reliability) [31]. An α level of ≥ 0.667 was considered the lowest limit of acceptable reliability [32], and values ≥ 0.8 were considered a good level of reliability [31]. Pairs of rater-generated codes assigned to each sample item were used to generate a bootstrap sample distribution of 1000 using an available macro for SPSS [30]. Doing so produced 95% confidence intervals for Krippendorff's α, allowing for an evaluation of the extent to which the ICF and IPC, serving as common classification frameworks for questionnaire items, yielded the same data among raters within a margin of error [26]. Where Krippendorff's α was determined for agreement on ICF codes alone, 'full item codes' that included multiple ICF codes applied to a single item were used for analysis. Rater-generated ICF codes were also included in the analysis. Multiple ICF codes assigned to individual items were re-listed in alphanumeric order (where applicable) prior to analysis. As a result, two items presenting the same concepts would be identically classified although they may differ in item perspective.

*Coding efficiency* was calculated as the number of unique framework codes required to classify sample items. The number of framework codes required to classify items varied depending on the number of unique concepts presented and/or the number of permutations and combinations of item perspectives found in questionnaire items. ICF and IPC framework codes and rater-generated codes were each considered in the analysis of coding efficiency.

*Content overlap detection* (%) was calculated as the percent of items that were assigned a classification code identical to a code assigned to at least one other item within the sample. The amount of 'shared' content overlap is important to consider when contrasting questionnaires, scales or subscales based on similarities or differences in the content presented. Given that individual item classifications may include ≥ 2 ICF categories, each ICF code assigned to an individual item was used to detect content overlap among the ICF codes assigned to all other sample items. Content overlap detection was further examined by determining the amount of shared content detected by only the five most frequently utilized classification codes.

## Results

### Classification capacity

**Fig. 3:**
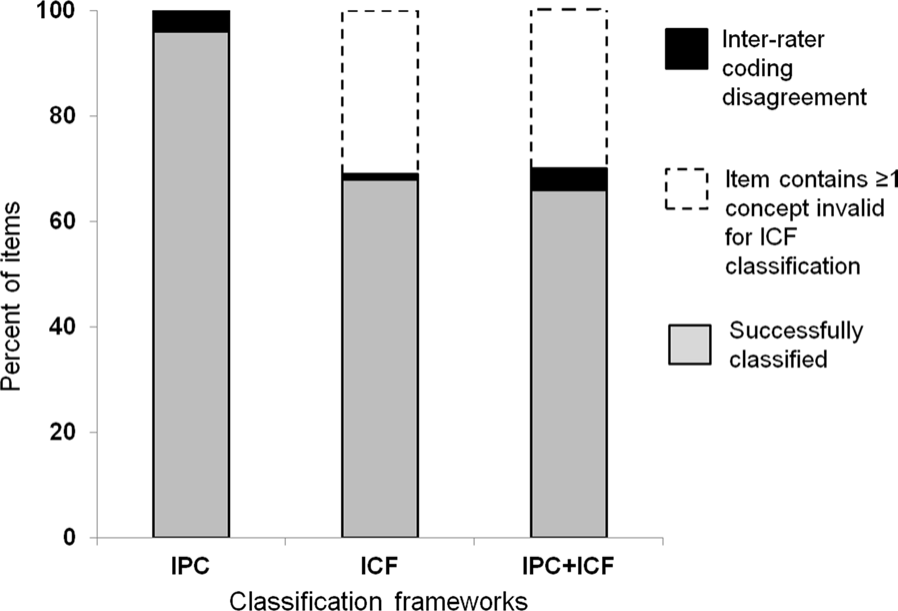
Classification capacity. Lightly shaded stacks indicate the percent of sample items that were successfully classified using IPC and ICF frameworks. Percent items unsuitable for ICF classification are denoted by the unshaded stacks. Percent of items remaining unresolved due to inter-rater coding disagreements are shown in black

Using the IPC framework, 100% of the 159 pooled items were successfully classified, and inter-rater coding agreement was 97%. In contrast, 69% of items were successfully classified using the ICF framework, and inter-rater coding agreement was 99%. For the remaining 31% of items that presented with one or more concepts unsuitable for ICF classification, 'rater-generated' ICF codes were assigned to each item. When composite item codes were generated using both the ICF and IPC frameworks, 66% of items were successfully classified, and inter-rater coding agreement was 97%. Accordingly, classification capacity was 97% using the IPC framework, 68% using the ICF framework, and 63% when both frameworks were used in conjunction (see Fig. 3). The classification capacity of composite item codes was restricted mainly due to item concepts being unsuitable to classification using the ICF framework rather than inter-rater coding disagreement.

### Reliability

Krippendorff's α was 0.97 (95% CI 0.94–0.99) for item classifications generated using the IPC framework. When ICF classifications assigned to each item were analyzed (without inclusion of IPC coding), Krippendorff's α was 0.99 (95% CI 0.97–1.00). Composite item codes yielded an inter-rater agreement at the level of Krippendorff's α of 0.97 (95% CI 0.95–0.99).

### Coding efficiency

**Fig. 4:**
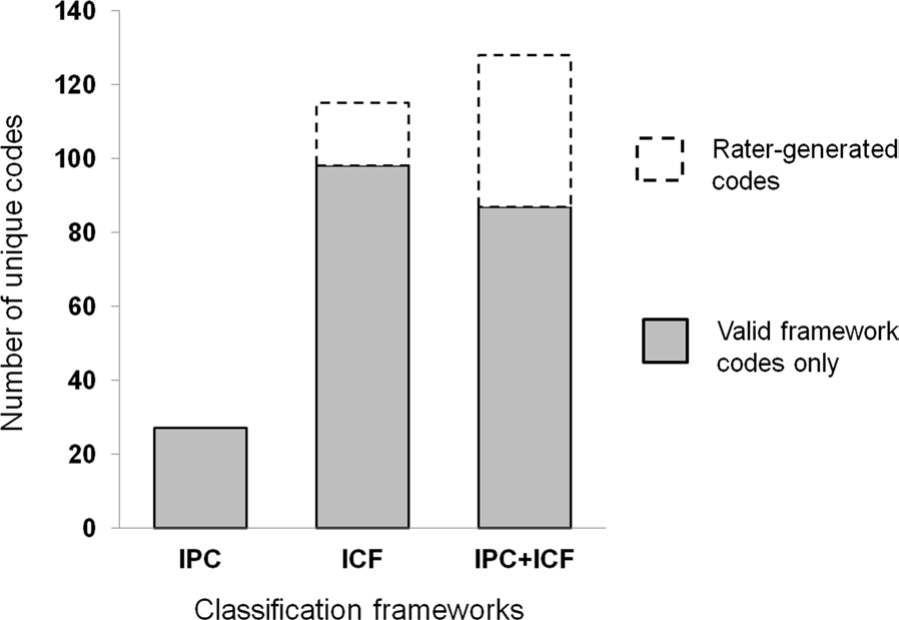
Coding efficiency. Total column height represents the number of unique codes required to classify sample items. Shaded stacks indicate the number of valid framework codes applied, and non-shaded stacks represent the number of item codes that contained at least one 'rater-generated' ICF category

A total of 26 unique IPC codes were used to classify 100% of sample items and a total of 97 unique ICF codes were used to successfully classify 69% of sample items. Accordingly, the creation of 17 unique rater-generated codes was necessary to classify the remaining 31% of items that were unsuitable for ICF classification. A total of 88 unique composite item codes were used to classify 66% of sample items, and an additional 41 unique composite item codes were 'rater-generated' ICF categories that were required to classify the remaining 34% of items (see Fig. 4). Thus, coding efficiency was 26 for the IPC framework, 114 for the ICF framework, and 129 for composite item codes combining the IPC and ICF framework when rater-generated ICF coding was taken into account.

### Content overlap detection

**Fig. 5:**
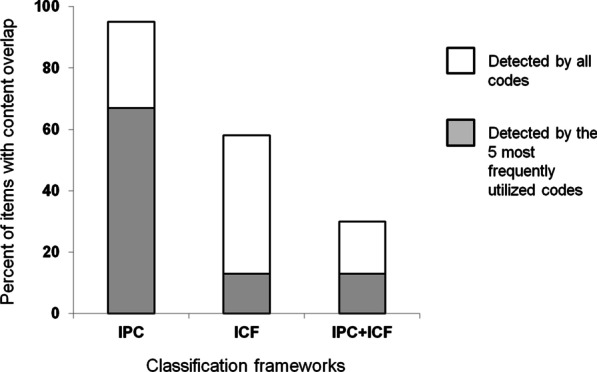
Content overlap detection. Total column heights indicate the percent of sample items found to share identical coding with at least one other item. Shaded stacks represent the percent of sample items with content overlap for the 5 most frequently used classification codes within each framework

Among sample items, 95% were assigned an IPC code that was identical to at least one other item (see Fig. 5). The 5 most frequently used IPC codes had content overlap among 67% of the sample items. In the absence of IPC classification, 58% of valid ICF codes appeared in at least one other item classification with the mean number of concepts per item being 1.7. The 5 most frequently used ICF codes had content overlap among 13% of the sample items. Finally, 30% of the sample items were assigned composite item codes that were identical to that of at least one other item. The 5 most frequently used composite item codes had content overlap amongst 13% of the sample items.

## Discussion

This study demonstrates that a simple classification of perspective (IPC framework) provides complementary information to that achieved by ICF linking, and that the IPC and ICF classification systems have contrasting performance characteristics when used for coding item content. The contrasting performance characteristics are in line with the small number of categories within the IPC framework and its use of interaction terms in comparison to the ICF framework, which has a large number of codes arranged in a hierarchical structure. Both systems demonstrate high inter-rater reliability, with differences between item classifications reflecting in the different approaches to item coding by the ICF and IPC frameworks. The ICF framework required the use of more than 4X the number of categories than the IPC framework, with the capacity for classifying 28% fewer items. Moreover, the ICF detected content overlap less frequently than the IPC framework, especially when the top 5 most frequently used classification codes were considered. This finding can be explained by the level of detail provided by each framework; the ICF provides greater detail on discrete content than what is provided by the IPC framework. The increased precision of the ICF comes at the cost of certain concepts such as those that are vague, unclear, or poorly defined as being unclassifiable with the ICF framework. Conversely the IPC framework may overestimate overlap since it provides broader classification.

A unique aspect of the IPC is that it classifies the type of judgment made by the respondent by providing item perspective. This is complementary to the focus on specific content taken by ICF which focuses on the aspect of disability. Item perspective can be important for understanding how items perform since respondents with the same level of disability may have very different emotional responses to that disability. This aspect of item content is more clearly identified by the IPC framework. For this reason, it is important to consider the IPC and ICF framework together, as they can provide discrepant, yet complementary information. When used in conjunction, these two frameworks yielded item classification codes that were detailed and multidimensional in their description of item content. However, increased detail is achieved with more complexity, which might make it more challenging to interpret the data.

The IPC framework can be used in situations where the goal is to identify a QoL questionnaire that covers a wide range of domains such as biological, social and psychological domains. Additionally, the IPC framework includes the syntax necessary to determine how these concepts relate to one-another. For example, the impact of social well-being on psychological health, the impact of psychological health on biological functioning, etc. This resolution is not achieved using the ICF framework alone. Take the example of a questionnaire that has 6 questions where each questionnaire contains the same two ICF codes: d7100 (respect and warmth in relationships) and b4200 (increased blood pressure), and both are coded identically. One cannot determine the differences between the 6 items using the ICF framework alone however, adding the use of the IPC framework the differences between the items can be revealed: (1) a rational appraisal of the impact of d7100 on b2400, (2) a rational appraisal of the impact of b2400 on d7100, (3) an emotional appraisal of the impact of d7100 on b2400, (4) an emotional appraisal of the impact of b2400 on d7100, (5) a rational appraisal of d7100 or b2400, and (6) an emotional appraisal of d7100 or b2400.

Similar to the findings of others [5, 14, 16], we found that *classification capacity* of the ICF was limited with abstract concepts such as 'quality of life', 'general health', and 'health state' which are concepts that are characterized as being subjective, vague, or poorly-defined. Such concepts may vary substantially in how they are defined by different raters. Abstract concepts not classifiable using the ICF framework were found in 31% of the sampled HRQoL items, but this percentage might be substantially lower among questionnaires that measure more narrowly-defined constructs [33]. Since the ICF was neither conceptually nor instrumentally designed to deal with abstract concepts, the classification capacity of this framework may vary according to the attributes of constructs included in questionnaires.

Classification capacity of the IPC framework was impacted by four inter-rater disagreements that occurred due to a lack of consensus on the amount of contextual information to be considered, and inconsistencies in determining which concepts were considered important to classify. These disagreements might suggest insufficiencies in the IPC framework and the available classification instructions [9], or may simply be indicative of poorly worded/focused items, or differences in the way content was interpreted. Coding disagreements have also occurred for these very same reasons in investigations that classified PROs using different ICF classification methods [4, 14]. On the other hand, application of the 'IPC-specific' ICF classification instructions proposed by Rosa [9] may have provided the necessary structure to achieve a high degree of clarity and consensus on these issues as evidenced by the high level of agreement that occurred when item content was linked with the ICF framework. However, given that few systems are universally applied without any errors we interpret our rater agreement as being excellent. Rater agreement reported in other studies has been variable and may range from 62 to 93% (or Cohen’s kappa from 0.67 to 1.00) depending on the number of raters, the amount of experience raters have in applying the ICF, and the components and chapter levels of ICF categories used for units of analysis [5, 14, 33, 39, 40]. Regarding classification capacity of composite item codes, it was found that classification restrictions occurred due to item concepts being unsuitable to classification using the ICF framework rather than inter-rater coding disagreement.

An especially high level of inter-rater agreement was achieved when assigning codes to items using the classification frameworks (α > 0.97). This may be attributable to the fact that both raters were involved in the development of the IPC, had extensive experience using the ICF as a stand-alone content classification tool, and through the development process spent a considerable amount of time coding together and discussing item classifications. Outside the context of this study, the high level of coding agreement achieved may not be representative in situations where raters have less experience with classification using either framework, or where classification occurs independently. However, the limited number of categories in the IPC framework may enhance potential for increasing reliability between raters, assuming the categories can be distinctly described. IPC content classification rules, whether employed alone or in conjunction with the ICF, have yet to be tested with regard to their reliability in the hands of different researchers. The reliability of the classification process may differ by increasing the number of raters, using raters from different professional backgrounds [5], or using a different sample of questionnaires. Additionally, it is important to consider that reports of reliability among ICF coders typically use statistics that are not readily applicable to interpretation, such as percent agreement. One major limitation of percent agreement is that it does not account for chance agreement, and high percent agreement becomes progressively unlikely as more categories are available for coding [30, 32]. Another frequently used statistic, Cohen’s kappa, is capable of accounting for chance agreement, however different variants in calculation are applied across study designs depending on the number of raters and attributes of coding data generated [5, 34–38]. Furthermore, Cohen’s kappa operates under the assumption that raters make their coding decisions independently, requiring that no rater has influence on another rater [41]. These assumptions were not upheld in this study, as discussion and collaboration were used in the development of the coding system. Observers were given the opportunity to come to agreement on classifications. Based on this, interpretation of reliability using Krippendorff's α is more applicable to this study. Krippendorff's α is also a statistic that accounts for chance agreement and can be used with any type of coding data generated from any number of raters [32].

Differences in *coding efficiency* among the IPC and ICF frameworks were attributable to the level of specification used when classifying item content. The IPC classified multidimensional attributes of items, not just the concepts contained within them, and only according to a few fundamental attributes. As a result, only 26 unique IPC codes were assigned to the pool of sample items. The framework did not possess the capacity to distinguish between highly specific attributes of similarly classified concepts. In contrast, the ICF classified only 1 dimension of item content (i.e. only the concepts presented) but was able to differentiate among numerous highly-specific conceptual attributes. Thus, the ICF was able to discern among many different concepts that are classified under the umbrella of a single IPC category. Although the IPC classifies a greater number of dimensions of item content, the enhanced concept specification and preciseness of description achieved by the ICF resulted in the use of a disproportionately large number of codes. The description of item content achieved from composite item codes was higher than that of the ICF alone, as the addition of item perspective to ICF coding allowed for the discernment of differences among items that would otherwise be similarly classified.

The amount of *content overlap* generated from the item classification process is necessary to consider when comparing measures based on similarities and differences. Even though the IPC classifies multiple attributes of item content and not just the concepts presented, content overlap was more readily detected than when items were classified using the ICF framework. This result can be explained due to the inherent nature of the IPC framework where its focus is on classifying concepts based on a few fundamental attributes, whereas the ICF being more precise in concept classification, was able to detect differences among items based on highly specific attributes. Accordingly, content overlap was detected less frequently when ICF codes were assigned, but a more precise depiction of concepts was achieved. The usefulness of this property will vary according to the reason for classification. For example, when searching for redundant items, the ability of the IPC framework to more readily detect potential conceptual overlap might be advantageous. However, if information on a specific trait is required, the ICF framework might provide the necessary detail to establish whether content overlap exists beyond broad categories of overarching concept domains.

Referring to the content overlap identified using composite item codes, it may be reasonable to assume that identically coded items are similar and comparable to one another on a number of attributes. Nonetheless, it is important to exercise caution when content overlap is determined by: (1) IPC codes alone since similarly classified concepts are likely to differ based on specific attributes, or (2) ICF codes alone since the content of similarly classified items may differ according to the nature of relationships that occur among multiple concepts, as well as the manner in which they are appraised by respondents. Accordingly, the utility of composite item codes rests in their ability to comprehensively describe multiple attributes of item content. See Table 1 for a summary of the individual strengths and weaknesses of the IPC and ICF frameworks.

**Table 1:** Summary table depicting the individual strengths and weaknesses of the IPC and ICF frameworks

	Strengths	Weaknesses
ICF Framework	High inter-rater reliability Provides greater detail on discrete content Increased precision Able to discern among many different concepts that are classified under the umbrella of a single IPC category	Required the use of more than 4X the number of categories Detected content overlap less frequently Limited classification capacity: vague, unclear, or poorly defined concepts are unclassifiable Does not consider *item perspective (as an emotional or rationale decision) or the relationship between content within an item*
IPC Framework	High inter-rater reliability Considers *item perspective* Better classification capacity Classifies a greater number of dimensions of item content	May overestimate content overlap due to broader classification

## Limitations

There are a few study limitations that need to be considered. Firstly, item classification was conducted by only two raters, and both raters were intimately involved in the development of the IPC framework, potentially introducing bias into the results of item classification. However, rater agreement was also high on ICF linking which has more codes and potentially more sources of disagreement. Generic HRQoL questionnaires were selected to provide breadth. Nonetheless, it would be important to test both classification systems in using condition-specific QoL measures to provide greater generalizability. Since the purpose of this paper was to evaluate the two classification systems, the 156 items from 6 HRQoL questionnaires were pooled. This limited the ability to provide a cross-PRO measure comparison, but this was not the intended purpose of the paper. The ICF linking rules were updated in 2019 to include a different type of prespective rating and we did not include these. Future research should evaluate the application of these coding systems in other contexts, with updated linking rules and investigate in what ways they inform decision-making around development or evaluation of content validity of PRO measures.

## Conclusion

The IPC framework successfully classified a larger proportion of questionnaire items, required fewer categories to do so, and identified content overlap in a larger percentage of items compared to the ICF. The ICF provided specific information about the content of functioning, activity, and participation items but did not describe the nature of abstract constructs or relationships that occur among ≥ 2 concepts, nor was it capable of describing the manner in which each item was appraised by respondents. When frameworks were used in conjunction to form composite item codes the data yielded were complimentary, provided detailed description of concepts, and described multiple dimensions of item content. Utilization of the IPC framework, either alone or in conjunction with the ICF, may be useful for classifying questionnaire items and the data generated may assist in determining content validity of PRO measures.

## Data Availability

The datasets generated and/or analysed during the current study are not publicly available.

## Abbreviations

HRQoL: Health related quality of life
ICF: International Classification of Functioning, Disability and Health
IPC: Item perspective classification
PRO: Patient reported outcome measures
